# Income and Marital Status Interact on Obesity Among Black and White Men

**DOI:** 10.1177/1557988319829952

**Published:** 2019-02-15

**Authors:** Caryn N. Bell, Roland J. Thorpe

**Affiliations:** 1 Department of African American Studies, University of Maryland, College Park, MD, USA; 2 Hopkins Center for Health Disparities Solutions, Johns Hopkins Bloomberg School of Public Health, Baltimore, MD, USA; 3 Department of Health, Behavior, & Society, Johns Hopkins Bloomberg School of Public Health, Baltimore, MD, USA

**Keywords:** men of color, special populations, obesity, behavioral issues, marriage, psychosocial and cultural issues

## Abstract

Racial disparities in obesity among men are accompanied by positive associations between income and obesity among Black men only. Race also moderates the positive association between marital status and obesity. This study sought to determine how race, income, and marital status interact on obesity among men. Using data from the 2007 to 2014 National Health and Nutrition Examination Survey, obesity was measured as body mass index ≥30 kg/m^2^ among 6,145 Black and White men. Income was measured by percentage of the federal poverty line and marital status was categorized as currently, formerly, or never married. Using logistic regression and interaction terms, the associations between income and obesity were assessed by race and marital status categories adjusted for covariates. Black compared to White (OR = 1.19, 95% CI [1.03, 1.38]), currently married compared to never married (OR = 1.45, 95% CI [1.24, 1.69]), and high-income men compared to low income men (OR = 1.26, 95% CI [1.06, 1.50]) had higher odds of obesity. A three-way interaction was significant and analyses identified that income was positively associated with obesity among currently married Black men and never married White men with the highest and lowest probabilities of obesity, respectively. High-income, currently married Black men had higher obesity rates and may be at increased risk for obesity-related morbidities.

Obesity is defined as having a body mass index (BMI) greater than 30 kg/m^2^. Obesity rates are increasing among many demographic groups (Flegal, Carroll, Kit, & Ogden, 2012; Flegal, Kruszon-Moran, Carroll, Fryar, & Ogden, 2016) with morbidity and mortality consequences (Bastien, Poirier, Lemieux, & Despres, 2014; Carnethon et al., 2017; Flegal, Kit, Orpana, & Graubard, 2013). Obesity is associated with higher mortality rates (Flegal et al., 2013), heart disease and other cardiovascular risk factors (Bastien et al., 2014), and some cancers (Bhaskaran et al., 2014). Race is an important factor in obesity rates in the United States (Bruce, Sims, Miller, Elliott, & Ladipo, 2007; Flegal et al., 2016; Hill et al., 2017; Ogden, Yanovski, Carroll, & Flegal, 2007; Seamans, Robinson, Thorpe, Cole, & LaVeist, 2015). Racial disparities in obesity between Black and White men have grown in recent years (Flegal et al., 2016; Ogden, Carroll, Kit, & Flegal, 2014).

Studies demonstrate a strong association between socioeconomic status (SES) and obesity among men (Bruce et al., 2007; Chang & Lauderdale, 2005; McLaren, 2007; Sanchez-Vaznaugh, Kawachi, Subramanian, Sanchez, & Acevedo-Garcia, 2009; Wardle, Waller, & Jarvis, 2002; Zhang & Wang, 2004). Though the predicted social gradient is observed among women, such that obesity rates decrease as SES increases, the associations differ among men (Bruce et al., 2007; Chang & Lauderdale, 2005; Sanchez-Vaznaugh et al., 2009; Zhang & Wang, 2004). Studies have either shown no protective association between higher SES and obesity or even one of increasing risk, particularly among Black men (Chang & Lauderdale, 2005; Griffith, Johnson-Lawrence, Gunter, & Neighbors, 2011; Sanchez-Vaznaugh et al., 2009; Zhang & Wang, 2004). Race appears to moderate the association between SES and obesity among men such that the associations differ between Black men compared to White men (Chang & Lauderdale, 2005; Griffith, Johnson-Lawrence, et al., 2011; Ogden et al., 2007; Zhang & Wang, 2004).

A 2018 study suggested that race moderates the association between marital status and obesity as well (Kroeger & Frank, 2018). Married and cohabiting men tend to have higher BMI or higher odds of being obese (Raley, Sweeney, & Wondra, 2015; Sobal, Hanson, & Frongillo, 2009; Sobal & Rauschenbach, 2003; Sobal, Rauschenbach, & Frongillo, 1992; Teachman, 2016). Though marriage rates are lower among Black men compared to White men (Raley et al., 2015), studies of race, BMI, and union status or marital transitions report that the positive effects of relationship status on increased obesity rates are stronger among Black men (Kroeger & Frank, 2018; Umberson, Liu, & Powers, 2009). Given that some studies demonstrate that income is positively associated with obesity among Black men (Chang & Lauderdale, 2005; Griffith, Johnson-Lawrence, et al., 2011; Sanchez-Vaznaugh et al., 2009; Zhang & Wang, 2004) as is being married or cohabiting (Kroeger & Frank, 2018; Umberson et al., 2009), it is important to assess the interrelationships between income, marital status, and obesity among men, and understand if the association varies between Black and White men.

Understanding how SES and marital status may operate synergistically to impact obesity rates among men is an important step to understanding how these social factors may operate differently for Black men compared to White men. These analyses may help identify subgroups that are at higher risk for obesity. The objective of this study is to determine whether the association between income and obesity differs by marital status and compare this relationship among Black men with White men. It is hypothesized that positive interactions between all three variables will be observed such that the highest obesity rates will be observed among married, high-income Black men.

## Methods

The National Health and Nutrition Examination Survey (NHANES) is an ongoing nationally representative survey of the health, functional, and nutritional statuses of the U.S. population. From the first data collection, each sequential series of this cross-sectional survey sampled the civilian noninstitutionalized population, with an oversample of low-income individuals, participants aged between 12 and 19 years, adults over the age of 60 years, Blacks, and Mexican Americans (Zipf et al., 2013). This survey used a stratified, multistage probability sampling design where data were collected in two phases. First, information regarding the participant’s health history, health behaviors, and risk factors was obtained during a home interview. Then, participants were invited to take part in a medical examination where they receive a detailed physical examination (Zipf et al., 2013). Because this study uses publicly available data, approval was not required. Data from 2007 to 2014 were combined to obtain a sufficient sample of Black men. The sample consisted of 6,145 non-Hispanic Black (*n* = 1,881) and non-Hispanic White men (*n* = 4,264) aged 20 years or older who completed the medical examination.

The dependent variable was obesity which was obtained by calculating respondents’ BMI from the height and weight measured in the medical examination. Respondents were considered obese if their BMI was ≥30 kg/m^2^. The independent variables included race, income, and marital status. Race was self-reported by asking respondents whether they were Hispanic/Latino or not, and then which racial group they belonged to. Non-Hispanic Blacks and Whites were included in these analyses. Income was measured by poverty-to-income ratio and categorized into three categories based on percentage of the federal poverty line (FPL): <200% FPL, 200%–400% FPL, ≥400% FPL. This measure of income accounts for household size. These particular cut-points were used to ensure that men with high income were included in the sample. Marital status was measured with a variable that included the following categories: currently married/cohabiting, formerly married (divorced/separated/widowed), never married. Covariates were self-reported and included the following: age, educational attainment, insurance status, self-rated health, current smoking, and physical inactivity. Age was measured continuously while the rest were measured dichotomously or with three or more categories. Insurance status measured whether or not the respondent had any health insurance. Educational attainment was categorized as: those who did not complete high school, high school graduates, or those who completed a General Education Development (GED) equivalent, those who completed some college or obtained an associate’s degree, and those who received a bachelor’s degree or more. Health status was measured dichotomously, comparing respondents reporting “fair” or “poor” against those reporting “excellent,” “very good,” or “good.” Survey respondents who currently smoke cigarettes every day or some days were considered current smokers. Physical inactivity represented survey respondents who do not participate in any moderate or vigorous physical activity.

The mean and proportional differences between race groups for demographic, SES, and other covariates were evaluated using Student’s *t*-test for continuous variables and chi-square tests for categorical variables. Associations between race, income, and marital status with obesity were assessed using logistic regression models that accounted for age (Model 1); age, education, insurance, fair/poor health, current smoking, and physical inactivity (Model 2); and included interaction terms between race, income, and marital status (Model 3). Wald tests were used to assess the three-way interaction and pairwise test were used to assess each interaction term combination. Predicted probabilities of obesity were calculated by combinations of race, marital status, and income. Marginal log odds were calculated to create predicted probabilities of obesity (presented as percentages) by combinations of race, marital status, and income adjusting for all covariates. Following the procedure recommended by the National Center for Health Statistics, all analyses used Taylor-linearization procedures for the complex multistage sampling design and a weight variable was created to account for the combining of multiple years of NHANES and using variables from the medical examination (Graham, 2015; Johnson et al., 2013a, 2013b). *P* values ≤.05 were considered statistically significant and all *t*-tests were two-sided. All statistical procedures were performed using Stata statistical software, Version 14 (StataCorp LP, College Station, TX).

## Results

Demographic and health differences by race among men were identified (see Table 1). Black men were younger, less likely to be insured, and less likely have bachelor’s degrees. Black men were more likely to report fair or poor health, be current smokers, and physically inactive. There were race differences for the distribution within marital status categories (*p* < .001). About half of Black men (*n* = 993, 51.2%) were currently married or living with a partner compared to almost 70% of White men (*n* = 2,851, 68.6%). Similar percentages of Black (*n* = 400, 18.1%) and White men (*n* = 697, 13.0%) were widowed, separated, or divorced. A third of Black men were never married (*n* = 488, 30.7%), while fewer than one in five White men were never married (*n* = 716, 18.4%). Race differences in income category distribution were also observed (*p* < .001). Fewer than one in four Black men were in the highest income group (*n* = 415, 21.8%), while almost half of White men had incomes ≥400% of the federal poverty line (FPL; *n* = 1,452, 45.7%). Half of Black men (*n* = 932, 49.4%) and a quarter of White men (*n* = 1,732, 25.9%) had incomes <200% FPL, but similar percentages of Black and White men were middle income (i.e., 200%–400% FPL). No difference in obesity (*p* = .079) was observed between Black men (*n* = 707, 37.5%) and White men (*n* = 1,453, 34.7%).

**Table 1.** Demographics, Socioeconomic Status, and Obesity Among Men, NHANES 2007–2014.

	Black	White	
	*N* = 1,881	*N* = 4,264	*p* value
Age (years), mean ± *SD*	44.3 ± 25.0	48.4 ± 24.0	<.001
Insured, *n* (%)	1,387 (68.8)	3,520 (85.5)	<.001
Education, *n* (%)			
Less than high school graduate	507 (24.6)	751 (11.9)	<.001
High school graduate	529 (28.5)	1,071 (23.7)	
Some college/associate’s degree	558 (31.2)	1,238 (30.6)	
Bachelor’s degree or more	287 (15.7)	1,204 (33.8)	
Fair/poor health, *n* (%)	458 (21.8)	759 (13.2)	<.001
Current smoker, *n* (%)	572 (31.3)	1,035 (21.7)	<.001
Physically inactive, *n* (%)	958 (47.3)	2,045 (42.0)	.006
Marital status, *n* (%)			
Currently	993 (51.2)	2,851 (68.6)	<.001
Formerly	400 (18.1)	697 (13.0)	
Never	488 (30.7)	716 (18.4)	
Income (federal poverty line), *n* (%)			
<200% FPL	932 (49.4)	1,732 (25.9)	<.001
200%–400% FPL	534 (28.8)	1,080 (28.4)	
≥400% FPL	415 (21.8)	1,452 (45.7)	
Obese, *n* (%)	707 (37.5)	1,453 (34.7)	.079

*Note*. Student’s *t-*test was used to determine the race difference for age. Chi-square tests were used to determine race differences for all other variables.

Associations between race, marital status, and income with obesity are displayed in Table 2. In Model 1, adjusting for age, Black race was associated with higher odds of obesity compared to White race (OR = 1.25, 95% CI [1.10, 1.44]), as was income 200%–400% FPL compared to income <200% FPL (OR = 1.22, 95% CI [1.03, 1.44]), and being never married was associated with lower odds (OR = 0.72, 95% CI [0.63, 0.82]) compared to being currently married. After adjusting further for education, insurance, fair/poor health, current smoking, and physical inactivity in Model 2, results were similar and Black race was associated with higher odds of obesity compared to being White (OR = 1.19, 95% CI [1.03–1.38]). Income was positively associated with obesity; the odds of obesity were higher among those with income between 200% and 400% FPL (OR = 1.29, 95% CI [1.09, 1.53]) and ≥400% FPL (OR = 1.26, 95% CI [1.06, 1.50]) compared to those with incomes <200% FPL. For both races, compared to men who were currently married, being never married (OR = 0.69, 95% CI [0.59, 0.81]) was associated with lower odds of obesity. In Model 3, the three-way interaction was assessed (*p* < .001), and odds of obesity for every race, income, and marital status combination were compared to White, low-income, currently married men. White, low-income, never married men had lower odds of obesity (OR = 0.49, 95% CI [0.35, 0.69]), as did Black, low-income, formerly married men (OR = 0.68, 95% CI [0.46, 0.99]). Higher odds of obesity were observed among White, middle-income, formerly married men (OR = 1.67, 95% CI [1.12, 2.49]) and Black, middle-income, currently married men (OR = 1.46, 95% CI [1.10, 1.96]).

**Table 2.** Association Between Race, Income, and Marital Status With Obesity Among Men, NHANES 2007–2014.

	Model 1	Model 2	Model 3
	OR (95% CI)	OR (95% CI)	OR (95% CI)
Black race	1.25 [1.10, 1.44]	1.19 [1.03, 1.38]	
Income			
<200% FPL	1.00	1.00	
200%–400% FPL	1.22 [1.03, 1.44]	1.29 [1.09, 1.53]	
≥400% FPL	1.06 [0.89, 1.27]	1.26 [1.06, 1.50]	
Marital status			
Currently	1.00	1.00	
Formerly	0.99 [0.80, 1.21]	0.96 [0.78, 1.20]	
Never	0.72 [0.63, 0.82]	0.69 [0.59, 0.81]	
Race × income × marital status			
White, <200% FPL, currently married			1.00
White, <200% FPL, formerly married			0.85 [0.61,1.18]
White, <200% FPL, never married			0.49 [0.35, 0.69]
White, 200%–400% FPL, currently married			1.06 [0.83, 1.36]
White, 200%–400% FPL, formerly married			1.67 [1.12, 2.49]
White, 200%–400% FPL, never married			0.84 [0.56, 1.25]
White, ≥400% FPL, currently married			1.09 [0.86, 1.38]
White, ≥400% FPL, formerly married			0.89 [0.57, 1.39]
White, ≥400% FPL, never married			0.86 [0.63, 1.17]
Black, <200% FPL, currently married			1.00 [0.74, 1.35]
Black, <200% FPL, formerly married			0.68 [0.46, 0.99]
Black, <200% FPL, never married			0.86 [0.59, 1.26]
Black, 200%–400% FPL, currently married			1.46 [1.10, 1.96]
Black, 200%–400% FPL, formerly married			0.87 [0.58, 1.28]
Black, 200%–400% FPL, never married			0.74 [0.47, 1.15]
Black, ≥400% FPL, currently married			1.68 [1.23, 2.30]
Black, ≥400% FPL, formerly married			1.35 [0.63, 2.88]
Black, ≥400% FPL, never married			1.23 [0.73, 2.05]

*Note.* Model 1 is adjusted for age. Model 2 adjusts for age, insurance, education, self-rated health, smoking status, and physical inactivity. Wald tests were performed for the three-way interaction term (*p* value <.001). The following pairwise comparisons of interaction terms were significant: Black, <200% FPL, never married–White, <200% FPL, never married (*p* = .011); Black, 200%–400% FPL, currently married–White, 200%–400% FPL, currently married (*p* = .023); Black, ≥400% FPL, currently married–White, ≥400% FPL, currently married (*p* = .005).

Table 3 demonstrates associations between income and obesity by race and marital status. Among Black men, a positive association between income and obesity was only observed among those who were currently married. Compared to those in the lowest income group, currently married Black men with the highest incomes had higher odds of obesity compared with never married Black men (OR = 1.51, 95% CI [1.05, 2.18]). A positive association between income and obesity was observed among White men, but only among those who were never married. Middle-income (OR = 1.72, 95% CI [1.07, 2.78]) and high-income (OR = 1.86, 95% CI [1.22, 2.82]), never married White men had higher odds of obesity than low-income White men who were never married.

**Table 3.** Associations Between Income and Obesity by Race and Marital Status Among Men, NHANES 2007–2014.

	Marital status		
	Never	Formerly	Currently
	OR (95% CI)	OR (95% CI)	OR (95% CI)
*Black*			
Income			
<200% FPL	1.00	1.00	1.00
200%–400% FPL	0.82 [0.52, 1.27]	1.04 [0.58, 1.88]	1.38 [1.00, 1.92]
≥400% FPL	1.19 [0.75, 1.91]	1.77 [0.69, 4.55]	1.51 [1.05, 2.18]
*White*			
Income			
<200% FPL	1.00	1.00	1.00
200%–400% FPL	1.72 [1.07, 2.78]	1.60 [0.96, 2.67]	1.09 [0.84, 1.40]
≥400% FPL	1.86 [1.22, 2.82]	0.87 [0.50, 1.52]	1.15 [0.87, 1.52]

*Note*. Models adjusted for age, insurance, education, self-rated health, smoking status, and physical inactivity.

**Figure 1.**
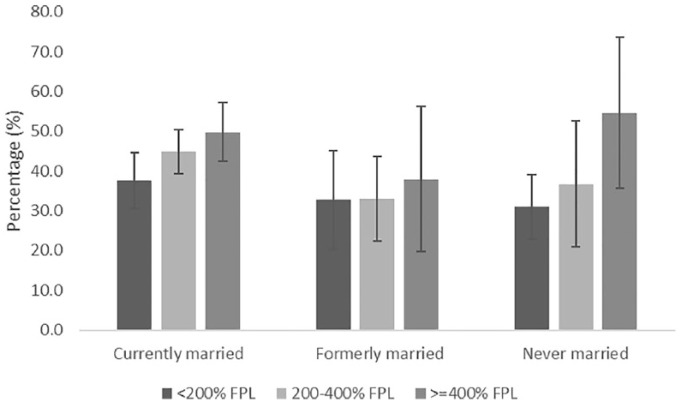
Predicted probabilities (95% confidence intervals) of obesity by income and marital status among Black men, NHANES 2007–2014. *Note*. Analyses adjusted for age, insurance, education, self-rated health, smoking status, and physical inactivity. Data represent marginal log odds. Statistically significant difference between lowest (reference) and highest income Black men who are currently married.

**Figure 2.**
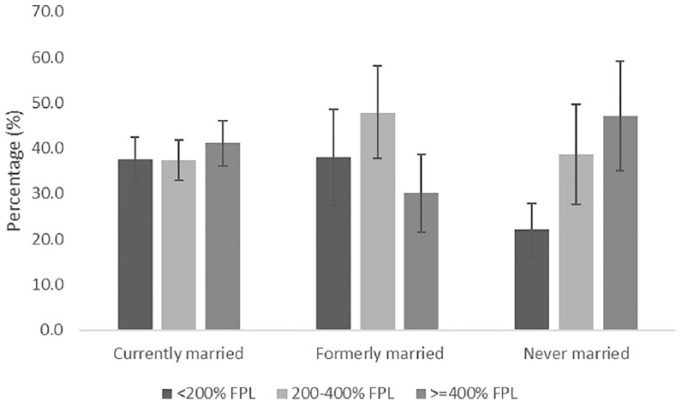
Predicted probabilities (95% confidence intervals) of obesity by income and marital status among White men, NHANES 2007–2014. *Note*. Analyses adjusted for age, insurance, education, self-rated health, smoking status, and physical inactivity. Data represent marginal log odds. Statistically significant difference between lowest (reference), middle, and highest income White men who were never married.

Figures 1 and 2 display predicted probabilities of obesity among Black and White men by income and marital status. The lowest probabilities of obesity observed among Black men were for those with low (27.9%) and middle income (28.8%), and who were formerly married, and among middle-income (27.9%) Black men who were never married. The highest probabilities of obesity were observed among currently married Black men with middle (45.6%) and high income (47.8%). Among White men, the lowest probability of obesity was observed among those with low incomes who were never married (20.3%). The highest probability of obesity among White men was observed in middle-income, formerly married men (44.8%).

## Discussion

This study sought to determine how race interacts with both income and marital status on obesity. The three-way interactions between race, income, and marital status were significant such that being Black and high income was associated with higher odds of obesity among currently married Black men, but not among those who were formerly or never married. For White men, there was a positive association between income and obesity only among those who were never married. Higher rates of obesity were observed among middle- and high-income, currently and formerly married Black men, as well as middle-income, formerly married White men. Obesity rates were lowest among low-income, never-married White men. These findings underscore the potential importance of social factors, namely income and marital status, in understanding disparities in obesity rates for Black and White men.

The results of the current study should be compared with previous literature which demonstrates mixed results. Previous studies have reported that race moderates the associations between marital status, union status, and marital transition with obesity among men (Griffith, Johnson-Lawrence, et al., 2011; Kroeger & Frank, 2018; Sobal et al., 2009). While there were no differences in obesity across income among formerly married White men, the results suggest that obesity rates were higher among formerly married men who were low or middle income compared to never married White men. There was no difference in obesity between never and formerly married high-income White men. Another study demonstrated that BMI decreased just before men divorced and after they became divorced (Syrda, 2017). Sobal et al. (2009) reported no association between marital status and obesity among Black men. However, a study by Griffith, Johnson-Lawrence, et al. (2011) identified that married Black men had higher obesity rates. A recent study of the transition to marriage demonstrated that among young adults, the effects of marriage were stronger on weight gain among Black men (Kroeger & Frank, 2018). This association was no longer statistically significant for when education was included in the model, unlike in the current study where racial differences in the associations between marital status, income, and obesity were still observed after adjusting for educational attainment.

The current study found that income was also positively associated with obesity among White men who were never married. Previous studies either identified no association between income and obesity among White men (Ogden, Lamb, Carroll, & Flegal, 2010; Ogden et al., 2017) or a relatively weak, but significant association (Chang & Lauderdale, 2005). Lower obesity rates among low-income men can be due to increased work-related physical activity. One study of leisure-time versus work-related physical activity reported that occupational activity is associated with lower likelihood of obesity (King et al., 2001). Lower income men may be more likely to be employed in fields like construction or maintenance that require physical activity (King et al., 2001). For White men, this explanation likely applies to the findings of the current study as the lowest obesity rates were observed in lower income, never married White men.

Continuing the comparison with previous literature, several studies have demonstrated that race moderates the effects of income on obesity among men such that higher income is associated with higher obesity rates among Black men (Chang & Lauderdale, 2005; Griffith, Johnson-Lawrence, et al., 2011; Ogden et al., 2007, 2010, 2017; Sanchez-Vaznaugh et al., 2009; Zhang & Wang, 2004). The results of the current study agree in that the odds of obesity increased with income among currently married men. For those who were never or formerly married, there was no association between income and obesity. The inverse association was observed among currently married men who, regardless of income, tend to have higher obesity rates than unmarried men. The explanation for the positive association between income and obesity may differ by race in that, for White men, it is possible that the positive association is due to work-related physical activity or other factors related to never being married. Among Black men, the positive association between income and obesity could combine with the effects of being currently married and result in the highest obesity rates among high-income, currently married Black men.

To explain the results of this study, theory on marital status and health should be applied. Most health outcomes improve, particularly among men, for those who are currently married (Wilson, 2012). Explanations for this include the Shared Risk Factor Model and the Health Investment Theory (Wilson, 2012). The Shared Risk Factor Model suggests that married couples pursue better health in light of the shared risk of poor health to the family unit, and these shared risk factors could be harmful or protective. The Health Investment Theory suggests that married people are able to achieve or maintain health due to increased efficiency in life tasks from having more than one person in the household. Obesity rates in the current study, as well as in previous studies (Raley et al., 2015; Sobal et al., 1992, 2009; Sobal & Rauschenbach, 2003; Teachman, 2016), tend to be higher among currently married Black and White men. This is consistent with the Marriage Market model (Syrda, 2017; Wilson, 2012), which suggests that lower BMI is observed among unmarried men because of perceived chance for mate selection and matching with women of similar body size. Simply stated, married men may not feel the need to maintain lower weight status because they are no longer a part of the marriage market.

For currently married Black men, these effects may combine with higher income and result in higher rates of obesity among the highest income, currently married men. Scholars have suggested that SES does not affect health among Blacks as it does among Whites (Pearson, 2008). Numerous studies have demonstrated the SES-health gradient is weaker or nonexistent among Blacks when compared to Whites. For Black men, higher income is associated with more discrimination (Colen, Ramey, Cooksey, & Williams, 2018; Williams, Priest, & Anderson, 2016), less leisure time (Griffith, Gunter, & Allen, 2011), and poorer neighborhoods (Reardon, Fox, & Townsend, 2015) that are more obesogenic than their White counterparts. This is due to interpersonal racism and structural racism that leads to less access to social goods needed for better health (Gee & Ford, 2011). Though the association between high income and obesity was not statistically significant among formerly married Black men, it suggests that those with high income tend to have higher obesity rates and these factors may contribute.

For currently married Black men, lower marriage rates among Blacks could play an important role. The inverse association between income and obesity among currently married Black men could reflect a social milieu where high-income, currently married men do not face the social pressures to maintain a healthy weight. Extending the Marriage Market theory of obesity (Syrda, 2017; Wilson, 2012), these men perceive that, in the marriage market, they are desirable due to higher income and those who are currently married do not perceive a need to maintain a healthy weight because the threat of going back into the marriage market is lower.

A competing explanation for high obesity rates among high-income, currently married Black men could be role strain (Griffith, Gunter, et al., 2011). For married Black men, the factors particular to higher-income Black men like discrimination may combine with the role strain of being a husband. Griffith, Gunter, et al. (2011) found that middle-class Black men report the importance of ensuring they meet the role of the provider, father, and spouse. The respondents also discussed how they prioritize their roles over health behaviors (Griffith, Gunter, et al., 2011). This could explain the higher obesity rates among currently and formerly married Black men with middle and high incomes. Regardless of the explanation, higher-income, currently and formerly married Black men with higher odds of obesity are at risk for obesity-related poor health outcomes such as cardiovascular disease. This higher risk is magnified among high-income, married Black men and it is possible that, because of higher income, attention to the health of this particular demographic is likely not a priority.

Though not statistically different from low- and high-income, formerly married men, white men who were middle-income and formerly married men had high obesity rates. These results suggest that income is an important factor in the association between obesity and marital status among White men as well. Other stressors may be associated with higher obesity rates among lower-income, formerly married White men. The particular combination of social factors in this group could lead to health behaviors such as potentially poorer diets or less physical activity which results in higher obesity rates. Though different health outcomes were examined, recent studies of diseases of despair in White, middle-aged adults suggest that social factors lead to poorer health among Whites (Case & Deaton, 2015). Similar factors could help explain obesity among middle-class, formerly married White men.

The study is strengthened by a nationally representative sample and the use of combined years of data. This allowed for enough high-income Black men in the analytic sample to enable sufficiently precise estimates (as indicated by the confidence intervals). The study also has some limitations. Normality when using *t*-tests for analyses could not be assessed because of the weighting of the data. Because the study is cross-sectional, the ability to determine causality was limited. Several studies on the association between marital status and obesity have examined transitions in marital status using a longitudinal design (Kroeger & Frank, 2018; Rauschenbach, Sobal, & Frongillo, 1995; Syrda, 2017; Teachman, 2016; Umberson et al., 2009). Income was measured categorically and results may have differed if measured continuously. Additional research to examine how race, income, and marital status interact on obesity over time would add to the literature.

## Conclusion

The findings from this study emphasize the importance of understanding how income and marital status operate independently and synergistically to influence obesity disparities in Black and White men. This work provides evidence that has the potential to lead to health promoting strategies among Black and White men. Understanding how other social determinants of health might affect obesity disparities in Black and White men is needed.

## References

[bibr1-1557988319829952] Bastien M. Poirier P. Lemieux I. Despres J.-P. (2014). Overview of epidemiology and contribution of obesity to cardiovascular disease. Progress in Cardiovascular Diseases, 56(4), 369–381. doi:10.1016/j.pcad.2013.10.016 24438728

[bibr2-1557988319829952] Bhaskaran K. Douglas I. Forbes H. dos-Santos-Silva I. Leon D. A. Smeeth L. (2014). Body-mass index and risk of 22 specific cancers: A population-based cohort study of 5.24 million UK adults. The Lancet, 384(9945), 755–765. doi:10.1016/s0140-6736(14)60892-8 PMC4151483 25129328

[bibr3-1557988319829952] Bruce M. A. Sims M. Miller S. Elliott V. Ladipo M. (2007). One size fits all? Race, gender and body mass index among US adults. Journal of the National Medical Association, 99(10), 1152–1158. 17987919 PMC2574391

[bibr4-1557988319829952] Carnethon M. R. Pu J. Howard G. Albert M. A. Anderson C. A. M. Bertoni A. G. … Yancy C. W. (2017). Cardiovascular health in African Americans: A scientific statement from the American heart association. Circulation, 136(21), E393–E423. doi:10.1161/cir.0000000000000534 29061565

[bibr5-1557988319829952] Case A. Deaton A. (2015). Rising morbidity and mortality in midlife among white non-Hispanic Americans in the 21st century. Proceedings of the National Academy of Sciences of the United States of America, 112(49), 15078–15083. doi:10.1073/pnas.1518393112 26575631 PMC4679063

[bibr6-1557988319829952] Chang V. W. Lauderdale D. S. (2005). Income disparities in body mass index and obesity in the United States, 1971–2002. Archives of Internal Medicine, 165(18), 2122–2128. doi:10.1001/archinte.165.18.2122 16217002

[bibr7-1557988319829952] Colen C. G. Ramey D. M. Cooksey E. C. Williams D. R. (2018). Racial disparities in health among nonpoor African Americans and Hispanics: The role of acute and chronic discrimination. Social Science & Medicine, 199, 167–180. doi:10.1016/j.socscimed.2017.04.051 28571900 PMC5673593

[bibr8-1557988319829952] Flegal K. M. Carroll M. D. Kit B. K. Ogden C. L. (2012). Prevalence of obesity and trends in the distribution of body mass index among US adults, 1999–2010. Journal of the American Medical Association, 307(5), 491–497. doi:10.1001/jama.2012.39 22253363

[bibr9-1557988319829952] Flegal K. M. Kit B. K. Orpana H. Graubard B. I. (2013). Association of all-cause mortality with overweight and obesity using standard body mass index categories: A systematic review and meta-analysis. JAMA: Journal of the American Medical Association, 309(1), 71–82. doi:10.1001/jama.2012.113905 23280227 PMC4855514

[bibr10-1557988319829952] Flegal K. M. Kruszon-Moran D. Carroll M. D. Fryar C. D. Ogden C. L. (2016). Trends in obesity among adults in the United States, 2005 to 2014. JAMA: Journal of the American Medical Association, 315(21), 2284–2291. doi:10.1001/jama.2016.6458 27272580 PMC11197437

[bibr11-1557988319829952] Gee G. C. Ford C. L. (2011). Structural racism and health inequities: Old issues, new directions. Du Bois Review: Social Science Research on Race, 8(1), 115–132. doi:10.1017/s1742058x11000130 25632292 PMC4306458

[bibr12-1557988319829952] Graham G. (2015). Disparities in cardiovascular disease risk in the United States. Current Cardiology Reviews, 11(3), 238–245. doi:10.2174/1573403x11666141122220003 25418513 PMC4558355

[bibr13-1557988319829952] Griffith D. M. Gunter K. Allen J. O. (2011). Male gender role strain as a barrier to African American men’s physical activity. Health Education & Behavior, 38(5), 482–491. doi:10.1177/1090198110383660 21632436 PMC4381925

[bibr14-1557988319829952] Griffith D. M. Johnson-Lawrence V. Gunter K. Neighbors H. W. (2011). Race, SES and obesity among men. Race and Social Problems, 3(4), 298–306. 10.1007/s12552-011-9051-5 PMC4104793 25057330

[bibr15-1557988319829952] Hill S. E. Bell C. Bowie J. V. Kelley E. Furr-Holden D. LaVeist T. A. Thorpe R. J. (2017). Differences in obesity among men of diverse racial and ethnic background. American Journal of Men’s Health, 11(4), 984–989. doi:10.1177/1557988315580348 PMC5675329 25862694

[bibr16-1557988319829952] Johnson C. L. Paulose-Ram R. Ogden C. L. Carroll M. D. Kruszan-Moran D. Dohrmann S. M. Curtin L. R. (2013a). National health and nutrition examination survey: Analytic guidelines, 1999–2010. Vital and Health Statistics. Series 2, Data Evaluation and Methods Research, 1–24. 25090154

[bibr17-1557988319829952] Johnson C. L. Paulose-Ram R. Ogden C. L. Carroll M. D. Kruszan-Moran D. Dohrmann S. M. Curtin L. R. (2013b). National health and nutrition examination survey: Analytic guidelines, 2011–2012. Retrieved from https://www.cdc.gov/nchs/data/nhanes/analytic_guidelines_11_12.pdf 25090154

[bibr18-1557988319829952] King G. A. Fitzhugh E. C. Bassett D. R. McLaughlin J. E. Strath S. J. Swartz A. M. Thompson D. L. (2001). Relationship of leisure-time physical activity and occupational activity to the prevalence of obesity. International Journal of Obesity, 25(5), 606–612. doi:10.1038/sj.ijo.0801583 11360141

[bibr19-1557988319829952] Kroeger R. A. Frank R. (2018). Race-ethnicity, union status, and change in body mass index in young adulthood. Journal of Marriage and Family, 80(2), 444–462. doi:10.1111/jomf.12454 29773921 PMC5950716

[bibr20-1557988319829952] McLaren L. (2007). Socioeconomic status and obesity. Epidemiologic Reviews, 29, 29–48. doi:10.1093/epirev/mxm001 17478442

[bibr21-1557988319829952] Ogden C. L. Carroll M. D. Kit B. K. Flegal K. M. (2014). Prevalence of childhood and adult obesity in the United States, 2011–2012. Journal of the American Medical Association, 311(8), 806–814. doi:10.1001/jama.2014.732 24570244 PMC4770258

[bibr22-1557988319829952] Ogden C. L. Fakhouri T. H. Carroll M. D. Hales C. M. Fryar C. D. Li X. Freedman D. S. (2017). Prevalence of obesity among adults, by household income and education — United States, 2011–2014. MMWR: Morbidity and Mortality Weekly Report, 66(50), 1369–1373. doi:10.15585/mmwr.mm6650a1 29267260 PMC5751581

[bibr23-1557988319829952] Ogden C. L. Lamb M. M. Carroll M. D. Flegal K. M. (2010). Obesity and socioeconomic status in adults: United States, 2005–2008. Hyattsville, MD: National Center for Health Statistics Retrieved from https://www.cdc.gov/nchs/data/databriefs/db50.pdf

[bibr24-1557988319829952] Ogden C. L. Yanovski S. Z. Carroll M. D. Flegal K. M. (2007). The epidemiology of obesity. Gastroenterology, 132(6), 2087–2102. doi:10.1053/j.gastro.2007.03.052 17498505

[bibr25-1557988319829952] Pearson J. (2008). Can’t buy me whiteness: New lessons from the Titanic on race, ethnicity, and health. Du Bois Review: Social Science Research on Race, 5(1), 27–47.

[bibr26-1557988319829952] Raley R. K. Sweeney M. M. Wondra D. (2015). The growing racial and ethnic divide in U.S. marriage patterns. The Future of Children, 25(2), 89–109. 27134512 10.1353/foc.2015.0014 PMC4850739

[bibr27-1557988319829952] Rauschenbach B. Sobal J. Frongillo E. A. (1995). The influence of change in marital-status on weight change over one-year. Obesity Research, 3(4), 319–327. doi:10.1002/j.1550-8528.1995.tb00157.x 8521148

[bibr28-1557988319829952] Reardon S. F. Fox L. Townsend J. (2015). Neighborhood income composition by household race and income, 1990–2009. The ANNALS of the American Academy of Political and Social Science, 660(1), 78–97.

[bibr29-1557988319829952] Sanchez-Vaznaugh E. V. Kawachi I. Subramanian S. V. Sanchez B. N. Acevedo-Garcia D. (2009). Do socioeconomic gradients in body mass index vary by race/ethnicity, gender, and birthplace? American Journal of Epidemiology, 169(9), 1102–1112. doi:10.1093/aje/kwp027 19299405

[bibr30-1557988319829952] Seamans M. J. Robinson W. R. Thorpe R. J. Cole S. R. LaVeist T. A. (2015). Exploring racial differences in the obesity gender gap. Annals of Epidemiology, 25(6), 420–425. doi:10.1016/j.annepidem.2015.03.010 25887701 PMC4433605

[bibr31-1557988319829952] Sobal J. Hanson K. L. Frongillo E. A. (2009). Gender, ethnicity, marital status, and body weight in the United States. Obesity, 17(12), 2223–2231. doi:10.1038/oby.2009.64 19300431

[bibr32-1557988319829952] Sobal J. Rauschenbach B. S. (2003). Gender, marital status, and body weight in older U.S. adults. Gender Issues, 21(3), 75–94.

[bibr33-1557988319829952] Sobal J. Rauschenbach B. S. Frongillo E. A. (1992). Marital-status, fatness and obesity. Social Science & Medicine, 35(7), 915–923. doi:10.1016/0277-9536(92)90106-z 1411692

[bibr34-1557988319829952] Syrda J. (2017). The impact of marriage and parenthood on male body mass index: Static and dynamic effects. Social Science & Medicine, 186, 148–155. doi:10.1016/j.socscimed.2017.05.033 28615139

[bibr35-1557988319829952] Teachman J. (2016). Body weight, marital status, and changes in marital status. Journal of Family Issues, 37(1), 74–96. doi:10.1177/0192513x13508404 26778872 PMC4714799

[bibr36-1557988319829952] Umberson D. Liu H. Powers D. (2009). Marital status, marital transitions, and body weight. Journal of Health and Social Behavior, 50(3), 327–343. doi:10.1177/002214650905000306 19711809 PMC3149893

[bibr37-1557988319829952] Wardle J. Waller J. Jarvis M. J. (2002). Sex differences in the association of socioeconomic status with obesity. American Journal of Public Health, 92(8), 1299–1304. doi:10.2105/ajph.92.8.1299 12144988 PMC1447234

[bibr38-1557988319829952] Williams D. R. Priest N. Anderson N. B. (2016). Understanding associations among race, socioeconomic status, and health: Patterns and prospects. Health Psychology, 35(4), 407–411. doi:10.1037/hea0000242 27018733 PMC4817358

[bibr39-1557988319829952] Wilson S. E. (2012). Marriage, gender and obesity in later life. Economics & Human Biology, 10(4), 431–453. doi:10.1016/j.ehb.2012.04.012 22795874

[bibr40-1557988319829952] Zhang Q. Wang Y. (2004). Trends in the association between obesity and socioeconomic status in U.S. adults: 1971 to 2000. Obesity Research, 12(10), 1622–1632. doi:10.1038/oby.2004.202 15536226

[bibr41-1557988319829952] Zipf G. Chiappa M. Porter K. S. Ostchega Y. Lewis B. G. Dostal J. (2013). National health and nutrition examination survey: Plan and operations, 1999–2010. Vital and Health Statistics. Ser. 1, Programs and Collection Procedures, 1–37. 25078429

